# Outbreak of Invasive *Serratia marcescens* among Persons Incarcerated in a State Prison, California, USA, March 2020–December 2022

**DOI:** 10.3201/eid3013.230801

**Published:** 2024-04

**Authors:** Amanda Kamali, Donna Ferguson, Heather Dowless, Nancy Ortiz, Rituparna Mukhopadhyay, Cassandra Schember, Rawni Lunsford, Justine Hutchinson, Marlena Scherer, John Crandall, Heidi Bauer, Alexander Yu, Akiko Kimura

**Affiliations:** California Correctional Health Care Services, Elk Grove, California, USA (A. Kamali, H. Dowless, J. Hutchinson, M. Scherer, H. Bauer);; California Department of Public Health, Richmond, California, USA (A. Kamali, N. Ortiz, R. Mukhopadhyay, C. Schember, J. Crandall, A. Yu, A. Kimura);; Monterey County Public Health Laboratory, Salinas, California, USA (D. Ferguson, R. Lunsford);; Centers for Disease Control and Prevention, Atlanta, Georgia, USA (N. Ortiz, C. Schember)

**Keywords:** Serratia marcescens, bacteria, nosocomial infections, persons using injection drugs, infectious diseases, prevention and control, prisons, California, United States

## Abstract

*Serratia marcescens* is an environmental gram-negative bacterium that causes invasive disease in rare cases. During 2020–2022, an outbreak of 21 invasive *Serratia* infections occurred in a prison in California, USA. Most (95%) patients had a history of recent injection drug use (IDU). We performed whole-genome sequencing and found isolates from 8 patients and 2 pieces of IDU equipment were closely related. We also identified social interactions among patients. We recovered *S. marcescens* from multiple environmental samples throughout the prison, including personal containers storing Cell Block 64 (CB64), a quaternary ammonium disinfectant solution. CB64 preparation and storage conditions were suboptimal for *S. marcescens* disinfection. The outbreak was likely caused by contaminated CB64 and propagated by shared IDU equipment and social connections. Ensuring appropriate preparation, storage, and availability of disinfectants and enacting interventions to counteract disease spread through IDU can reduce risks for invasive *Serratia* infections in California prisons.

*Serratia marcescens*, a gram-negative environmental bacterium (*1*,*2*), is an opportunistic pathogen that in rare cases causes invasive diseases, including bacteremia and endocarditis (*1*,*3*–*7*). Reported outbreaks have been linked to contaminated environmental sources, such as water, soap, intravenous fluids, and compounded drugs (*8*–*16*) in nosocomial settings (*17*–*19*). Invasive *S. marcescens* infections have occurred among persons who inject drugs (*5*,*6*,*20*–*22*). Given the high prevalence of injection drug use (IDU) in prisons and lack of access to sterile needles (*23*–*25*), risks for transmission of bloodborne pathogens are higher than among the general population (*25*). Cell Block 64 (CB64) solution, produced by California Prison Industry (CALPIA, https://www.calpia.ca.gov), is a quaternary ammonium concentrate (https://catalog.calpia.ca.gov/custom/assets/Files/view-current-sds-information-16.pdf) used as the primary disinfectant in prisons in California, USA. However, *S. marcescens* can survive in improperly prepared disinfection solutions, including quaternary ammonium disinfectants (*18*,*19*,*26*). 

We describe a multiyear outbreak of invasive *S. marcescens* infections driven by widespread environmental contamination, improperly prepared and maintained disinfection solution, IDU, and social connections at a California state prison. Prison A is a maximum-security state prison housing ≈3,000 male incarcerated persons. In October 2020, the primary hospital affiliated with prison A notified the California Correctional Health Care System (CCHCS) that multiple incarcerated persons had been admitted with invasive *S. marcescens* infections. CCHCS, Monterey County Public Health Laboratory (MCPHL), and California Department of Public Health (CDPH) began a multidisciplinary investigation to identify additional cases, determine risk factors for infection, and provide recommendations for mitigation and prevention. This project was determined to be nonresearch by the Centers for Disease Control and Prevention because it involved public health surveillance. 

## Materials and Methods

### Epidemiologic Investigation

We defined a case-patient as a person diagnosed with an invasive *S. marcescens* infection who resided at prison A for ≥1 month before symptom onset during January 1, 2020–December 31, 2022. We defined infections as invasive if occurring at normally sterile body sites or in a case-patient manifesting critical illness with severe soft tissue infection. We reviewed patient hospitalization and prison medical records, including social histories, for IDU and other risk-elevating behaviors. We interviewed patients using a standardized questionnaire that included questions about cell cleaning practices, IDU, and other risk factors. 

### Environmental Investigation

Prison A public health and infection control, CCHCS public health, CDPH, and MCPHL staff evaluated the water system and cleaning practices and procedures at prison A. In 2020 and 2021, MCPHL tested water from different sources at prison A, including holding tanks and wells. MCPHL also tested sinks and communal showers, faucets in patients’ cells, personal items, hand-rinsate from a cellmate, 2 syringes used for injecting drugs, objects used for mixing, storing, or applying disinfectant, dilution machines, reused containers, and commercial bottles.

### Laboratory Investigation

MCPHL streaked swabs onto *Serratia* CHROMagar (https://www.chromagar.com) MacConkey and blood agar plates and incubated them in brain heart infusion broth for up to 5 days. Needles and syringes were placed directly into brain heart infusion broth. Cultures with growth were subcultured on CHROMagar plates. Liquids, including water, disinfectant cleaning solutions, and rinsates, were filtered onto 47 mm 0.45 μm–pore sized mixed cellulose ester membranes and placed onto CHROMagar plates. MCPHL forwarded *S. marcescens* isolates to CDPH Center for Laboratory Science Microbial Diseases Laboratory for whole genome sequencing (WGS) using the validated in-house protocol with Illumina MiSeq (https://www.illumina.com) (Appendix) (*27*). 

## Results

### Epidemiologic Investigation

**Figure 1:**
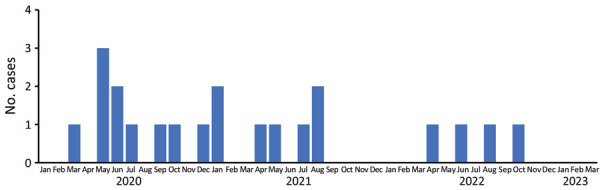
Epidemiologic curve of patients hospitalized with invasive *Serratia marcescens* infections at prison A, by sampling month of positive isolate, California, USA, January 2020–March 2023.

As of December 2022, we had identified 21 cases: 17 identified during March 2020–August 2021 and 4 during April–October 2022 (Figure 1). All 21 case-patients were hospitalized and recovered; however, 1 patient later died of a cause unrelated to *S. marcescens*. Median patient age was 44 years (range 22–66 years). We grouped patients by race/ethnicity as non-Hispanic White (9 [43%]), non-Hispanic Black (2 [10%]), Hispanic (8 [38%]), or other (2 [10%]). Diagnoses were not mutually exclusive and included bacteremia in 11 (52%) patients; endocarditis in 2 (10%); epidural abscess in 9 (43%); osteomyelitis in 6 (29%); pseudoaneurysm in 1 (5%); and soft tissue infections in 4 (19%), including 2 (10%) with muscle abscess (Table 1). Of the nonbacteremic patients, 2 had polymicrobial cultures, including viridans streptococci (*1*), *Staphylococcus aureus* (*2*), and *Raoutella panticola* (*1*). 

**Table 1:** Demographic data and other characteristics of 21 patients infected with invasive *Serratia marcescens* at prison A, California, USA, 2020–2022*

Characteristic	Value
Median age, y (range)	44 (22–66)
Race and ethnicity	
White	9 (43)
Black	2 (10)
Hispanic	8 (38)
Other	2 (10)
*Serratia* diagnosis†	
Bacteremia	11 (52)
Endocarditis	2 (10)
Epidural abscess	9 (43)
Osteomyelitis	6 (29)
Pseudoaneurysm	1 (5)
Severe soft tissue infection	4 (19)
Type of injection drug used,† n = 21	
Heroin	18 (86)
Suboxone	12 (57)
Methamphetamine	8 (38)
Opiates (by hospital urine toxicology screen)	5 (24)

*Values are no. (%) patients except as indicated. †Not mutually exclusive.

Twenty (95%) patients had a history of IDU <6 months before infection and one >6 months before infection. Of patients with recent IDU, 18/20 (86%) had injected heroin, 12 (57%) suboxone, and 8 (38%) methamphetamines. Among patients who had urine toxicology performed at admission, 4/9 were positive for opiates. Nine patients reported consuming >1 drug; 5 patients used 2 and 4 patients used 3 drugs. Of patients interviewed, 5/16 (31%) used CB64 to clean IDU equipment. Of PWID patients, 9/21 (43%) were enrolled in the prison A substance use disorder treatment (SUDT) program before *S. marcescens* infection occurred.

**Figure 2:**
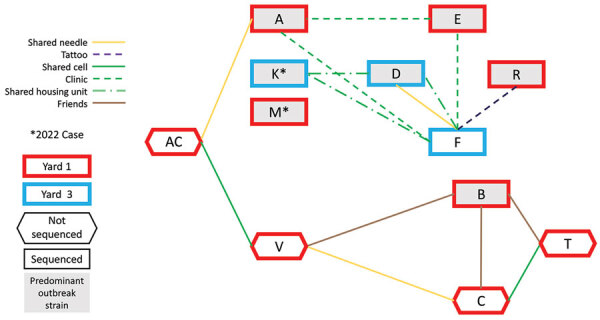
Social network analysis of patients and whole genome sequencing results for patients hospitalized with invasive *Serratia marcescens* infections at prison A, California, USA, January 2020–March 2023. All patients were identified in 2021, except patients K and M, identified in 2022. Patients A, B, D, E, R, K, and M all had isolates in the predominant outbreak strain. Patients D, F, and K were in yard 3, all others in yard 1. Patients C, T, and V did not have isolates available for sequencing. Patient AC had a *S. marcescens* infection in 2019 outside of the outbreak period; however, he had multiple social connections with case-patients and so is included in this figure. Patient F shared a housing unit with D and K, was in the clinic at the same time as A and E, reported sharing needles with D, and might have been tattooed by R. Patient D also shared a housing unit with K. Patient A was in the clinic the same time as E and reported sharing a needle with AC. Patient V shared a cell with AC, was friends with D, and reported sharing needles with C. Patient T shared a cell with C and was friends with B. Patients B and C were also friends.

**Figure 3:**
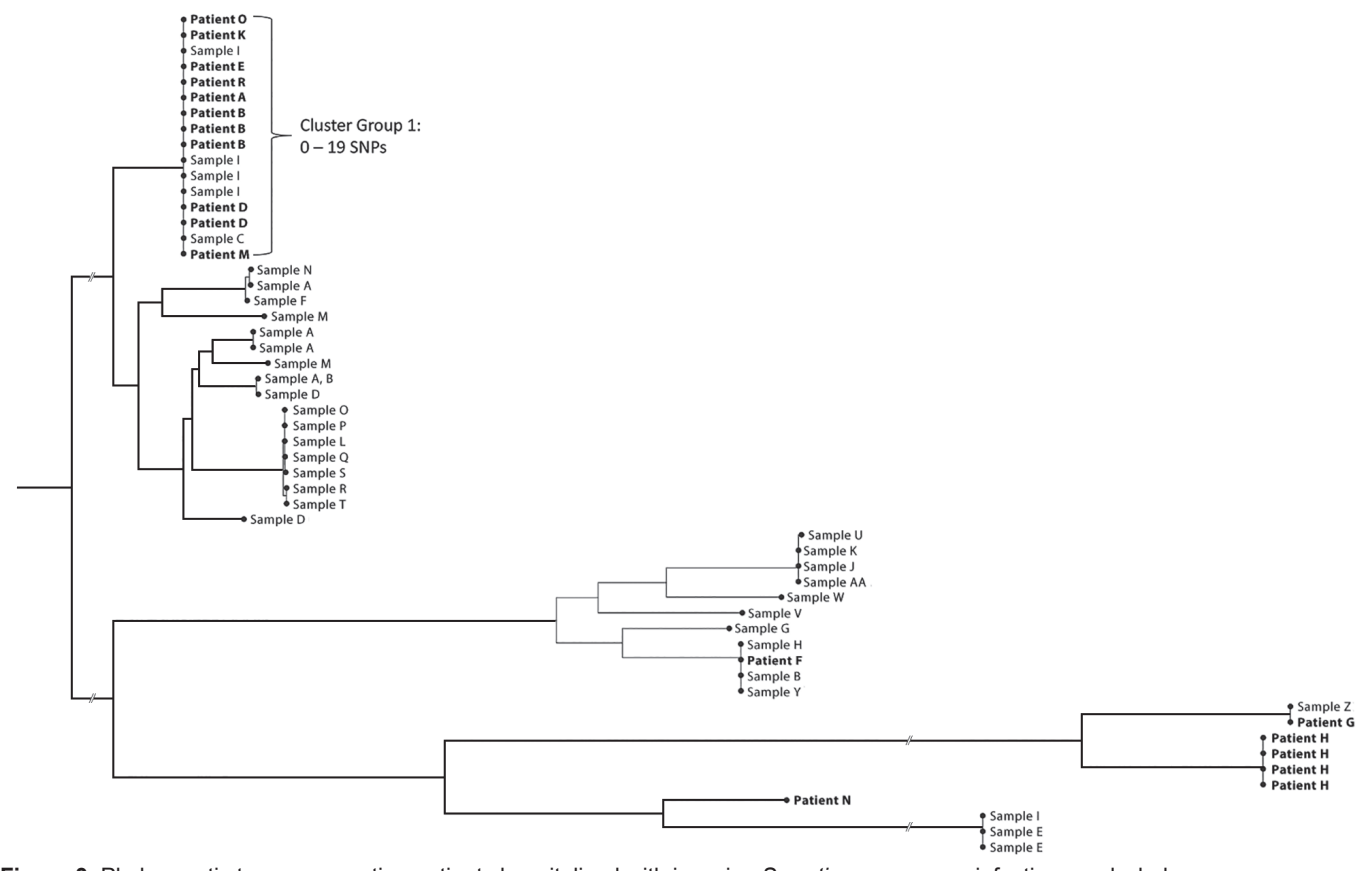
Phylogenetic tree representing patients hospitalized with invasive *Serratia marcescens* infections and whole-genome sequencing for environmental and clinical isolates at prison A, California, USA, January 2020–March 2023. The predominant outbreak cluster included patients A, B, D, E, K, M, O, and R and environmental samples C (needle/syringe) and sample I (nasal spray bottle) from patient D. These sequences had 0–19 single-nucleotide polymorphism (SNP) differences. Patient F, sample B (coffee cup) found in patient D’s cell, sample H (hand rinsate) from the cellmate of patient D, and sample Y (doorway swab) from the cell occupied at different times by both patient A and AC are grouped together within a 11–17 SNP range.

Although some patients resided throughout the 4 physically separated yards at the facility, 11 (52%) were housed in yard 1; 2 patients in other yards at time of illness onset had previously been housed in yard 1. Interviews identified social connections among >9 patients. We used WGS to identify the predominant *S. marcescens* outbreak strain as the cause of infection in 6 (66%) patients and a different strain in 1 patient; we had no isolates available for 4 patients (Figures 2, 3). Among patients who revealed social connections, 6 shared needles, 4 shared cells, 3 had attended the urgent care clinic at the same time, and 2 might have shared tattoo needles (Figure 2). Patient AC, diagnosed with an invasive *S. marcescens* infection at a different prison in 2019 and later transferred to prison A, was found to have multiple social connections with patients identified in 2020 and beyond but we did not include him in the outbreak cohort (Figure 2). 

### Environmental Investigation

**Figure 4:**
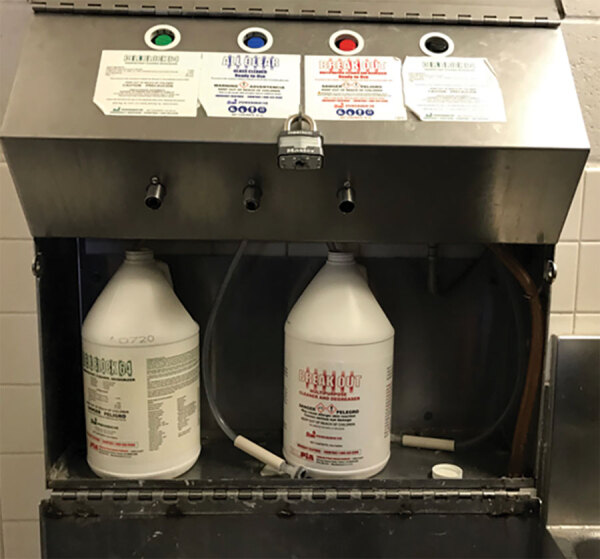
Device calibrated to dilute Cell Block 64 solution and other cleaners to correct concentrations. Device pictured shows dangling tubing touching the machine surface, a possible route of contamination in outbreak of invasive *Serratia marcescens* infections at prison A, California, USA, January 2020–March 2023.

Inspection of the potable water system at prison A did not identify any deficiencies or areas of concern, and we found large-volume water samples negative for *Serratia.* Each housing unit has a machine for diluting the CB64 solution (Figure 4). Machines in multiple units had exposed tubing touching the machine surface, maintenance schedules were not documented, and dilution of CB64 occurred in large containers outside the dilution machines. Prison A allowed incarcerated persons to keep CB64 in their cells after the COVID-19 pandemic started. Some incarcerated persons described using their own repurposed containers (e.g., shampoo bottles) to scoop diluted CB64 from the large containers. 

### Laboratory Investigation

Eleven patients had isolates available for WGS; 8 (73%) patients, including 3 identified in 2022, had isolates that differed from one another by 0–19 single-nucleotide polymorphisms (SNPs) on WGS. Those isolates clustered within the predominant strain group (Figure 3). Of 152 environmental samples collected and analyzed, 27 (18%) were positive for *S. marcescens*, including a needle and syringe combination (sample I) and a reused nasal spray bottle (sample C) storing methamphetamines from patient D (Table 2). Both specimens matched the predominant outbreak strain (Figure 3). The *S. marcescens* isolate from patient F grouped within 11–17 SNPs with isolates from a coffee cup (sample B) found in patient D’s cell, hand-rinsate (sample H) from patient D’s cellmate, and a doorway swab (sample Y) from a cell occupied at different times by patients AA and AC (Table 2; Figure 3). All other isolates differed from the predominant strain by thousands of SNPs (Figure 3). We sequenced multiple isolates from some samples. Samples from all unopened bottles of CB64 tested negative for *S. marcescens*. 

**Table 2:** Environmental specimens positive for *Serratia marcescens* associated with patients hospitalized with invasive *Serratia marcescens* infections at prison A, California, USA, January 2020–March 2023

Identifier	Sample description	Sample location, yard no.	Matched a patient isolate
A	Swab of water pooled in Cell Block 64 solution dilution machine tubes	2	No
B	Coffee from plastic cup	3	Yes
C	Nasal spray bottle	3	Yes, patients B and D
D	Scrub pad 1: porter closet	1	No
E	Scrub pad 2: used to clean cell	1	No
F	Scrub pad 3: beside toilet	1	No
G	Shower floor swab	1	No
H	Hand rinsate of cellmate to patient D (sterile saline)	3	No
I	Used needle or syringe 1	1	Yes, patients A and B
J	Water and laundry detergent from body wash bottle	1	No
K	Cleaner stored in hand sanitizer bottle	1	No
L	Diluted cell block 64 solution in spray bottle	1	No
M	Mop bucket	1	No
N	Cell Block 64 solution stored in shampoo bottle	1	No
O	Cell Block 64 solution stored in chili sauce bottle	2	No
P	Cell Block 64 solution stored in coffee container	2	No
Q	Empty bottle, used to store Cell Block 64 solution	1	No
R	Diluted breakout from trash can 1	1	No
S	Doorway 4 floor swab	1	No
T	Doorway 3 floor swab	1	No
U	Drinking water in bottle	2	No
V	Breakout from container originally used to store Cell Block 64 solution	2	No
W	Doorway 2 floor swab	1	No
Y	Doorway 1 floor swab	1	Yes
Z	Plastic sports drink bottle, used to store water	1	No
AA	Plastic bottle used as urinal	1	No
AB	Diluted breakout from trash can 2	1	No

## Discussion 

During March 2020–December 2022, a total of 21 persons incarcerated at prison A required hospitalization for invasive *Serratia* infections. Factors contributing to this outbreak included widespread environmental contamination with *Serratia,* including in CB64, the sole disinfectant used within the prison, and complex social networks that involved IDU.

Of note, 5 environmental samples that tested positive for *Serratia* were associated with diluted CB64. CB64 is used throughout California state prisons as a disinfectant because it is less caustic than other disinfectants (e.g., bleach). Quaternary ammonium compounds like CB64 have previously been linked to outbreaks (*18*,*19*,*26*). Prison A had documented nonadherence to CB64 manufacturer dilution and storage protocols. In addition, incarcerated persons stored diluted CB64 in cells after the COVID-19 pandemic began, a change in procedure occurring at approximately the same time as initial cases. Repeatedly finding *Serratia* in CB64 indicates that improper use and storage of the disinfectant likely contributed to the spread. 

The invasive nature of the *Serratia* infections, including manifestations such as bacteremia and severe soft-tissue infection, suggests introduction of the bacteria directly into the bloodstream or soft tissues, highlighting the role of IDU in the prison outbreak. The predominant outbreak strain of *Serratia* was recovered from a needle obtained from 1 patient. In prisons, there is no access to new needles; some patients reported sharing needles, and most reported reusing needles multiple times themselves. Some patients reported using CB64 to clean their needles. 

In August 2021, prison A implemented mitigation measures, including extensive staff training, instituting maintenance logs, recalibrating dilution machines, ensuring regular changing of tubing in dilution devices, and providing dedicated bottles of CB64 for incarcerated persons to check out and return within 24 hours for in-cell cleaning. Additional education on IDU risks and SUDT (begun in 2020) were also provided to incarcerated persons. No new cases were identified until spring 2022, at which time lapses in staff and resident education on use, maintenance, and storage of CB64 solution and dilution devices were recognized.

WGS results for 3 patient isolates identified at the prison in 2022 were closely related to 2021 patient isolates, indicating that the predominant outbreak strain of *S. marcescens* persisted >1 year*.* Given the diversity of *S. marcescens* strains in the environment, the predominance of a single strain suggests the likely existence of a persistent, but unknown, nidus of the outbreak strain. A single contaminated drug or CB64 source is unlikely to account for the persistence. An incarcerated person colonized with this strain or an unrecognized fomite in the environment are possible sources. Although *S. marcescens* is not a normal part of human flora, colonization of skin and gut has been documented (*10*,*27*). In addition, the hand-rinsate from a patient’s cellmate yielded *S. marcescens*, indicating the potential for persistence on skin. After identification of additional cases in 2022, intervention included reeducating staff and incarcerated persons on proper use of CB64, including performing dilution within dilution devices only, and education on risks for *S. marcescens* infection through IDU equipment. No further cases had been documented as of July 2023, 8 months after the last identified case. Additional education has been provided to institutions throughout the state (Appendix). 

One limitation of this study is that, given drug use is prohibited in prison, patients might have provided incomplete information regarding drug preparation and sharing, and therefore some common sources of drugs or drug equipment may not have been identified. In addition, only 2 needles or syringes were available for testing. A comprehensive environmental sampling survey of the entire prison population and structure was unfeasible, so we focused testing on areas where cases were identified. Additional sources of environmental contamination, including water sources such as cell toilet water and shower and sink drains and traps, where biofilm may have formed, were unable to be tested. A limited number of patient isolates from 2021 and 2022 were available for WGS; testing of all isolates might have further clarified patient connections. Patients might have been infected with >1 *S. marcescens* strain. Most environmental isolates positive for *S. marcescens* did not match patient strains, and so direct correlation between environmental contamination and patient illness was not possible. Finally, our investigation focused on invasive infections and excluded milder illness.

Beginning in January 2020, screening and referral for SUDT became available in California prisons to all newly incarcerated persons, those transitioning into the community, and patients with IDU-related complications (*28*). As of January 2022, >64,600 incarcerated persons had been screened for SUD and medication-assisted treatment provided to >22,500 patients, leading to a significantly decline in overdoses and infectious disease complications since the program started (*29*). 

After this outbreak, queries have identified additional cases of invasive *S. marcescens* infections in other California prisons. Similar concerns related to disinfection, including improper storage, device calibration, and usage, and IDU practices have been reported. Environmental mitigation through extensive cleaning and strict adherence to disinfectant guidelines might not eliminate all environmental sources of *Serratia* but might decrease the environmental microbial burden, thereby decreasing potential exposures to *S. marcescens* and other pathogens. IDU among incarcerated persons should be addressed through promotion of harm reduction practices and education, including access to appropriate disinfection supplies and sterile needles, and referral to SUDT programs. 

Appendix Additional information for study of invasive *Serratia marcescens* among persons incarcerated in state prison, California, USA, March 2020–December 2022.

## References

[1] Fisher RG. Serratia. In: Feigin RD, Cherry JD, Demmler-Harrison GJ, Kaplan SL, eds. Feigin and Cherry’s textbook of pediatric infectious diseases (6th edition). Philadelphia: W.B. Saunders, 2009. pp. 1563–7.

[2] Donnenberg MS. Enterobacteriaceae. Bennett JE, Dolin R, and Blaser MJ, eds. Mandell, Douglas, and Bennett’s principles and practice of infectious diseases, 8th edition. Philadelphia: W.B. Saunders, 2015. p. 2503–17.e5.

[3] Mahlen SD. *Serratia* infections: from military experiments to current practice. Clin Microbiol Rev. 2011;24:755–91. 10.1128/CMR.00017-11 21976608 PMC3194826

[4] Rana A, Rabbani NUA, Wood S, McCorkle C, Gilkerson C. A complicated case of vertebral osteomyelitis by *Serratia marcescens.* Cureus. 2020;12:e9002. 10.7759/cureus.9002 32775082 PMC7402549

[5] Sanchez KT, Johnson LB, Szpunar S, Saravolatz LD. A case of mixed *Serratia marcescens* and *Streptococcus mitis* endocarditis and review of the literature. Infect Dis Clin Pract. 2012;20:245–7. 10.1097/IPC.0b013e318242430c

[6] Schecter MC, Spicer JO, Aldrete SDM, Kraft CS. *Serratia marcescens* infectious endocarditis injection drug use, left-sided heart disease, and poor outcomes. Infect Dis Clin Pract. 2018;26:216–9. 10.1097/IPC.0000000000000614

[7] Hadid H, Usman M, Thapa S. Severe osteomyelitis and septic arthritis due to *Serratia marcescens* in an immunocompetent patient. Case Rep Infect Dis. 2015;2015:347652. 10.1155/2015/347652 26161276 PMC4486246

[8] Horcajada JP, Martínez JA, Alcón A, Marco F, De Lazzari E, de Matos A, et al. Acquisition of multidrug-resistant *Serratia marcescens* by critically ill patients who consumed tap water during receipt of oral medication. Infect Control Hosp Epidemiol. 2006;27:774–7. 10.1086/504445 16807859

[9] Civen R, Vugia DJ, Alexander R, Brunner W, Taylor S, Parris N, et al. Outbreak of *Serratia marcescens* infections following injection of betamethasone compounded at a community pharmacy. Clin Infect Dis. 2006;43:831–7. 10.1086/507336 16941362

[10] Cristina ML, Sartini M, Spagnolo AM. *Serratia marcescens* infections in neonatal intensive care units (NICUs). Int J Environ Res Public Health. 2019;16:610. 10.3390/ijerph16040610 30791509 PMC6406414

[11] Caggiano G, Triggiano F, Diella G, Apollonio F, Lopuzzo M, Mosca A, et al. A possible outbreak by *Serratia marcescens*: genetic relatedness between clinical and environmental strains. Int J Environ Res Public Health. 2021;18:9814. 10.3390/ijerph18189814 34574734 PMC8472797

[12] Archibald LK, Corl A, Shah B, Schulte M, Arduino MJ, Aguero S, et al. *Serratia marcescens* outbreak associated with extrinsic contamination of 1% chlorxylenol soap. Infect Control Hosp Epidemiol. 1997;18:704–9. 10.2307/30141511 9350463

[13] Henry B, Plante-Jenkins C, Ostrowska K. An outbreak of *Serratia marcescens* associated with the anesthetic agent propofol. Am J Infect Control. 2001;29:312–5. 10.1067/mic.2001.117043 11584257

[14] Ostrowsky BE, Whitener C, Bredenberg HK, Carson LA, Holt S, Hutwagner L, et al. *Serratia marcescens* bacteremia traced to an infused narcotic. N Engl J Med. 2002;346:1529–37. 10.1056/NEJMoa012370 12015392

[15] Boyce JM, Havill NL. In-use contamination of a hospital-grade disinfectant. Am J Infect Control. 2022;50:1296–301. 10.1016/j.ajic.2022.03.008 35307473

[16] Weber DJ, Rutala WA, Sickbert-Bennett EE. Outbreaks associated with contaminated antiseptics and disinfectants. Antimicrob Agents Chemother. 2007;51:4217–24. 10.1128/AAC.00138-07 17908945 PMC2167968

[17] de Boer MG, Brunsveld-Reinders AH, Salomons EM, Dijkshoorn L, Bernards AT, van den Berg PCM, et al. Multifactorial origin of high incidence of *Serratia marcescens* in a cardio-thoracic ICU: analysis of risk factors and epidemiological characteristics. J Infect. 2008;56:446–53. 10.1016/j.jinf.2008.04.001 18511122

[18] Rohit A, Suresh Kumar D, Dhinakaran I, Joy J, Vijay Kumar D, Kumar Ballamoole K, et al. Whole-genome-based analysis reveals multiclone *Serratia marcescens* outbreaks in a non-Neonatal Intensive Care Unit setting in a tertiary care hospital in India. J Med Microbiol. 2019;68:616–21. 10.1099/jmm.0.000947 30839251

[19] de Vries JJ, Baas WH, van der Ploeg K, Heesink A, Degener JE, Arends JP. Outbreak of *Serratia marcescens* colonization and infection traced to a healthcare worker with long-term carriage on the hands. Infect Control Hosp Epidemiol. 2006;27:1153–8. 10.1086/508818 17080370

[20] Peterson TC, Pearson C, Zekaj M, Hudson I, Fakhouri G, Vaidya R. Septic arthritis in intravenous drug abusers: a historical comparison of habits and pathogens. J Emerg Med. 2014;47:723–8. 10.1016/j.jemermed.2014.06.059 25282119

[21] Phadke VK, Jacob JT. Marvelous but Morbid: Infective endocarditis due to *Serratia marcescens.* [Baltim Md]. Infect Dis Clin Pract (Baltim Md). 2016;24:143–50. 10.1097/IPC.0000000000000360 27346925 PMC4917215

[22] Cooper R, Mills J. *Serratia* endocarditis. A follow-up report. Arch Intern Med. 1980;140:199–202. 10.1001/archinte.1980.00330140057018 6986128

[23] Bick JA. Infection control in jails and prisons. Clin Infect Dis. 2007;45:1047–55. 10.1086/521910 17879924

[24] Davis DM, Bello JK, Rottnek F. Care of incarcerated patients. Am Fam Physician. 2018;98:577–83. 30365288

[25] Hammett TM. HIV/AIDS and other infectious diseases among correctional inmates: transmission, burden, and an appropriate response. Am J Public Health. 2006;96:974–8. 10.2105/AJPH.2005.066993 16449578 PMC1470637

[26] Nakashima AK, Highsmith AK, Martone WJ. Survival of *Serratia marcescens* in benzalkonium chloride and in multiple-dose medication vials: relationship to epidemic septic arthritis. J Clin Microbiol. 1987;25:1019–21. 10.1128/jcm.25.6.1019-1021.1987 3298309 PMC269127

[27] Kozyreva VK, Truong CL, Greninger AL, Crandall J, Mukhopadhyay R, Chaturvedi V. Validation and implementation of Clinical Laboratory Improvements Act—compliant whole-genome sequencing in the public health microbiology laboratory. J Clin Microbiol. 2017;55:2502–20. 10.1128/JCM.00361-17 28592550 PMC5527429

[28] Jones SR, Amon M, Falvey C, Patrick K. *Serratia marcescens* colonising the gut. Lancet. 1978;1:1105. 10.1016/S0140-6736(78)90955-8 77407

[29] California Department of Corrections and Rehabilitation, California Correctional Health Care Services. Transforming substance use disorder treatment in California’s prison system. Impacts of the Integrated Substance Use Disorder Treatment Program 2019–2021, April 2022 [cited 2023 May 1]. https://cchcs.ca.gov/wp-content/uploads/sites/60/ISUDT/Impacts-ISUDT-Program2019-22.pdf

