# Supplementary material for: Outbreak of Invasive Serratia marcescens among Persons Incarcerated in a State Prison, California, USA, March 2020–December 2022
Source: Emerg Infect Dis. 2024 Apr;30(Suppl 1):S41–8. doi: 10.3201/eid3013.230801 (PMC10986826; doi:10.3201/eid3013.230801)
Supplement: Appendix — Additional information for study of invasive Serratia marcescens among persons incarcerated in state prison, California, USA, March 2020–December 2022. [file 23-0801-Techapp-s1.pdf]

Article DOI: https://doi.org/10.3201/eid3013.230801

EID cannot ensure accessibility for Appendix materials supplied by authors. Readers who have difficulty accessing Appendix content should contact the authors for assistance.

Outbreak of Invasive *Serratia marcescens* among Persons Incarcerated in a State Prison, California, USA, March 2020–December 2022

Appendix.

Appendix Table. Data on patient serosamples from study of persons incarcerated in a California state prison (BioProject no. PRJNA981498)

Patient identifier	Fastq file name	BioSample accession no.	Sequence read run no.
Patient F	PatientF	SAMN35676889	SRR24876887
Patient A	PatientA	SAMN35676882	SRR24876944
Patient E	PatientE	SAMN35676888	SRR24876888
Sample A	SampleA-2	SAMN35676907	SRR24876926
Sample A	SampleA-3	SAMN35676908	SRR24876925
Sample A	SampleA-1	SAMN35676906	SRR24876927
Patient I	PatientI	SAMN35676895	SRR24876939
Patient J	PatientJ	SAMN35676896	SRR24876938
Patient H	PatientH-1	SAMN35676891	SRR24876885
Patient H	PatientH-2	SAMN35676892	SRR24876942
Patient H	PatientH-3	SAMN35676893	SRR24876941
Patient H	PatientH-4	SAMN35676894	SRR24876940
Sample U	SampleU	SAMN35676937	SRR24876893
Sample O	SampleO	SAMN35676931	SRR24876900
Sample P	SampleP	SAMN35676932	SRR24876898
Patient B	PatientB-1	SAMN35676883	SRR24876943
Sample V	SampleV	SAMN35676938	SRR24876892
Sample M	SampleM-2	SAMN35676929	SRR24876902
Sample M	SampleM-1	SAMN35676928	SRR24876903
Sample D	SampleD-2	SAMN35676914	SRR24876918
Sample D	SampleD-1	SAMN35676913	SRR24876919
Patient B	PatientB-2	SAMN35676884	SRR24876932
Patient B	PatientB-3	SAMN35676885	SRR24876921
Sample E	SampleE-1	SAMN35676915	SRR24876917
Sample E	SampleE-2	SAMN35676916	SRR24876916
Sample I	SampleI-1	SAMN35676920	SRR24876912
Sample I	SampleI-2	SAMN35676921	SRR24876911
Sample I	SampleI-3	SAMN35676922	SRR24876909
Sample I	SampleI-4	SAMN35676923	SRR24876908
Sample F	SampleF	SAMN35676917	SRR24876915
Sample I	SampleI-5	SAMN35676924	SRR24876907
Sample N	SampleN	SAMN35676930	SRR24876901
Sample Q	SampleQ	SAMN35676933	SRR24876897
Patient S	PatientS	SAMN35676905	SRR24876928
Patient D	PatientD-1	SAMN35676886	SRR24876910
Patient D	PatientD-2	SAMN35676887	SRR24876899
Sample H	SampleH	SAMN35676919	SRR24876913
Sample B	SampleB	SAMN35676911	SRR24876922
Sample C	SampleC	SAMN35676912	SRR24876920
Patient R	PatientR	SAMN35676904	SRR24876929

Patient identifier	Fastq file name	BioSample accession no.	Sequence read run no.
Patient G	PatientG	SAMN35676890	SRR24876886
Sample W	SampleW	SAMN35676939	SRR24876891
Sample Y	SampleY	SAMN35676940	SRR24876890
Sample G	SampleG	SAMN35676918	SRR24876914
Sample R	SampleR	SAMN35676934	SRR24876896
Sample Z	SampleZ	SAMN35676941	SRR24876889
Sample S	SampleS	SAMN35676935	SRR24876895
Sample T	SampleT	SAMN35676936	SRR24876894
Sample AB	SampleAB	SAMN35676910	SRR24876923
Sample J	SampleJ	SAMN35676925	SRR24876906
Sample AA	SampleAA	SAMN35676909	SRR24876924
Sample K	SampleK	SAMN35676926	SRR24876905
Sample L	SampleL	SAMN35676927	SRR24876904
Patient N	PatientN	SAMN35676900	SRR24876934
Patient K	PatientK	SAMN35676897	SRR24876937
Patient L	PatientL	SAMN35676898	SRR24876936
Patient M	PatientM	SAMN35676899	SRR24876935
Patient P	PatientP	SAMN35676902	SRR24876931
Patient O	PatientO	SAMN35676901	SRR24876933
Patient Q	PatientQ	SAMN35676903	SRR24876930

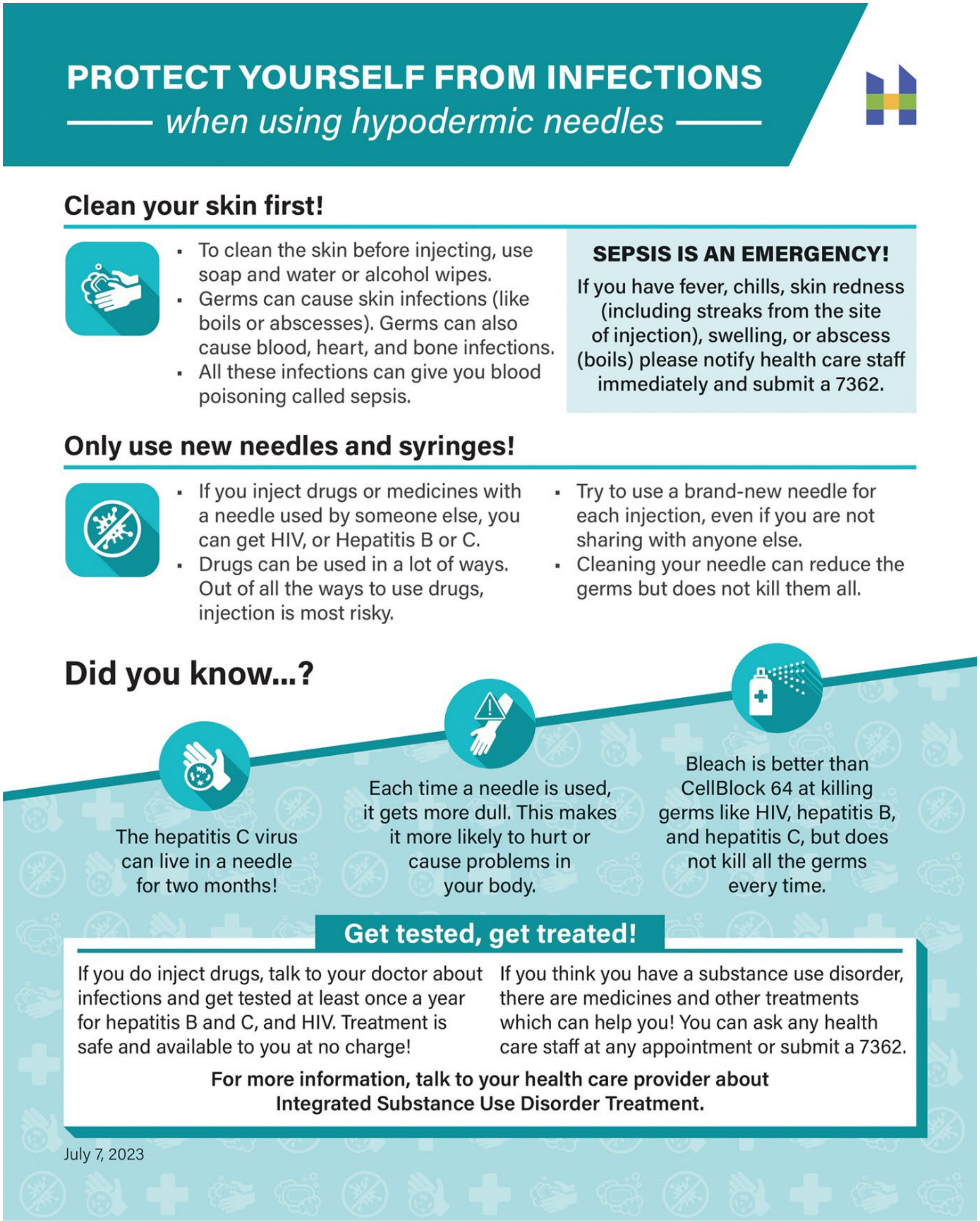

Appendix Figure. Injection safety poster from study of persons incarcerated in a California state prison.
